# Maple syrup urine disease decompensation misdiagnosed as a psychotic event

**DOI:** 10.1016/j.ymgmr.2022.100886

**Published:** 2022-06-18

**Authors:** Tomoyasu Higashimoto, Matthew T. Whitehead, Erin MacLeod, Danielle Starin, Debra S. Regier

**Affiliations:** a National Human Genome Research Institute, National Institutes of Health, Bethesda, MD, United States of America; b Division of Radiology, Children's National Hospital, Washington, DC, United States of America; c Division of Neuroradiology, Children's Hospital of Philadelphia, Philadelphia, PA, United States of America; d Department of Radiology Perelman School of Medicine, University of Pennsylvania, Philadelphia, PA, United States of America; e Children's National Rare Disease Institute, Washington, DC, United States of America

**Keywords:** Maple syrup urine disease, Branched-chain α-ketoacid dehydrogenase enzyme complex, Branched-chain amino acids, Transition of care, MSUD, maple syrup urine disease, BCAA, branch chain amino acids, ADHD, attention deficit hyperactivity disorder, CT, computed tomography, IEM, inborn errors of metabolism

## Abstract

Maple syrup urine disease (MSUD) is an autosomal recessive metabolic disease resulting in impaired or absent breakdown of branched-chain amino acids (BCAA) valine, isoleucine, and leucine. Classic MSUD often presents in post-natal periods, at times before newborn screening results, and is treated with a protein restricted diet supplemented with medical food and close follow up to prevent toxic buildup of blood leucine.

Acute episodes of decompensation are prevented by early recognition and treatment. Acute episodes of metabolic decompensation in patients with MSUD are medical emergencies that require immediate treatments as cerebral edema may lead to brain-stem compression resulting in death. As the early outcomes improve for MSUD patients, the long-term sequelae of chronic hyperleucemia are being elucidated and include cognitive impairment, mental health disorders, and movement disorders.

In this report we present an adult patient with MSUD with attention deficit, hyperactivity type (ADHD) and depression due to prolonged exposure to elevated leucine managed with community support services who presented to the emergency department with new onset of acute hallucinations. He was held in the emergency department awaiting involuntary commitment to a psychiatric facility and underwent psychiatric treatments for suspected new onset hallucinations without improvement. Upon notification of metabolic specialists and initiation of appropriate therapy of MSUD, his leucine level normalized rapidly with resolution of his acute psychosis.

This case describes the acute presentation of psychosis in the setting of long-term toxicity of leucine. This case also highlights the importance of transition of care, education and planning in patients with inborn errors of metabolism.

## 1 Introduction

Maple syrup urine disease (MSUD) is an autosomal recessive metabolic disease due to deficiency and/or impaired function of branched-chain α-ketoacid dehydrogenase enzyme complex. This results in impaired or absent breakdown of branched-chain amino acids (BCAA) valine, isoleucine, and leucine [1].

Classic MSUD often presents by postnatal days five to seven with poor feeding, lethargy, seizures, encephalopathy, and death if not treated [2]. Intermittent MSUD is a variant form of the classic disease that frequently presents later in childhood with developmental delay [2].

Acute treatment goal is to reduce leucine concentrations in blood rapidly. To achieve this goal the commonly utilized therapy includes but not limited to providing 1.5 to 3 times the estimated energy requirements as dextrose (50%–70%) and lipid (30%–50%), continuous insulin infusion, limiting the total protein intake (2.0–3.5 g/kg/day as BCAA-free amino acids) by utilization of formula that does not contain branched chain amino acids, isoleucine and valine supplements (20–120 mg/kg per day each) to titrate to plasma concentrations of 400–800 μmol/L. Renal replacement therapies using dialysis or hemofiltration should be considered and maybe appropriate for some patients [[2], [3], [4]].

During acute metabolic decompensations, cerebral edema leading to brain-stem compression may result in death. Acute episodes of metabolic decompensation in patients with MSUD are medical emergencies that require immediate treatment [3,4]. Acute decompensation in older individuals can include cognitive impairment, hyperactivity, sleep disturbances, hallucinations, mood swings, focal dystonia, choreoathetosis, and ataxia [5]. Acute decompensations events are not identified solely by symptoms, but also systematic monitoring, if possible, of leucine levels.

Long-term management of classic MSUD requires a protein-restricted diet with BCAA-free formula supplementation [6]. If diagnosed and treated early, prognosis is generally favorable although even patients who strictly adhere to dietary restrictions are likely to experience metabolic decompensations [[7], [8], [9]]. Acute episodes of decompensation are prevented by early recognition of symptoms and treatment of underlying insults that may lead to protein catabolism [4].

While early diagnosis and implementation of treatment may lead to favorable outcome, patients with MSUD develop neurological and neurocognitive dysfunction [[9], [10], [11], [12]]. Incidence of attention deficit hyperactivity disorder (ADHD) is reported in significant proportion of MSUD patients [9,11,12]. Longitudinal study of MSUD patients have shown that most patients develop depression, anxiety, and panic disorder by age 35 [9,11,12]. Diagnosis of anxiety and depression are five to ten times more likely to develop in those who were encephalopathic as newborns at the time of diagnosis [11]. Furthermore, prolonged neonatal encephalopathy is the single strongest predictor of neurocognitive dysfunction [9,11].

Here we report a 29-year-old male with MSUD who presented to the emergency department and was subsequently admitted to psychiatric unit due to suspected new onset hallucinations who underwent psychiatric treatments without improvement.

## 2 Case presentation

Patient is a 29-year-old with MSUD, ADHD, depression, and history of compression injury to optic nerve likely secondary to longstanding elevated leucine and cerebral edema. He was diagnosed with ADHD around age nine, and he was subsequently started on medical treatment for ADHD at nine. Depressed mood was noted when the patient was 12 years old. He has had intermittent evaluation/care by psychiatry in his adolescence, and he was treated with antidepressant and antipsychotic medication in is 20s. In the years prior to this acute episode, he was receiving community-based mental health support and had stopped medications successfully. When he was 21 years old, he reported changes in his vision. He was subsequently evaluated by ophthalmologist who noted evidence of optic nerve compression injury. While not able to be confirmed, it was suspected that this was secondary to his history of cerebral edema.

The patient has experienced multiple decompensation events requiring multiple hospitalizations and had poor metabolic control throughout adulthood. His baseline leucine level often ranges between 500 and 800 μmoles/L. During his most recent decompensation event, patient was evaluated in a community-based emergency department and subsequently admitted to a psychiatric holding unit for concern for new onset hallucinations.

Per his medical records, he presented to the hospital complaining that he has not been sleeping well. Unfortunately, he was a poor historian secondary to his decompensation, and the hospital he presented to had limited knowledge of his medical history, due to the hospitals who knew him being on bypass secondary to the COVID-19 pandemic. While he was issued emergency letters in the past, he did not have the letter with him at the time of transport to the hospital. He did have a card in his wallet from his metabolic geneticist. This card requested that any emergency service team contact the on-call team and was laminated. When asked, he shared he did not give them the card, since they took his wallet during his time in the holding unit.

During the initial evaluation, he reported that he hallucinated a few times prior to presenting to the hospital when he was folding towels and thought he was holding snakes. His initial encounter is reported as “he seems somewhat disengaged and not interested in help. It is unclear whether he is homeless and has secondary gain versus having a true psychiatric acute issue.” His initial laboratory evaluation included normal CBC, CMP, negative urine toxicology screen, negative influenza/COVID-19, and urine analysis notable for presence of ketones. His bicarbonate level on the chemistry panel was 25 (normal: 22–29). Plasma lactate and ammonia levels were not obtained at that time.

Upon admission to the hospital patient was out of bed banging on walls screaming and rushing into other patients' rooms and acting threateningly toward patients and staff. Attempt was made to redirect several times, but it was unsuccessful. He received sedative medications per protocol. His subsequent evaluation reported “patient more awake after initial medications”. At this time, patient reported that he has MSUD and reported that he has been hearing voices. Emergency room physician reported “with reassessment after medications, I have low suspicion that maple syrup urine disease is the drive of his symptoms. He is able to answer questions more appropriately at this time. He notes that he brought himself in.”

His medical records notes that the patient was unsteady when he walked and stumbled at times. Plasma amino acids, urine analysis, and head CT was obtained for further evaluation. His urine was notable for presence of ketones, but his head CT was reported as normal (reading by adult radiologist). Patient received multiple sedative medications due to agitations while awaiting placement. His chemical sedations included Haldol and Ativan (day 1), Benadryl, Haldol, and Ativan (day 2), and Haldol and Ativan (day 3). On the second day of his admission, he was started on daily Olanzapine treatment for psychosis and on the third day started on Sertraline treatment for depression.

His primary metabolic geneticist was notified regarding the patient four days after his initial presentation to the emergency room. He was evaluated in person by the metabolic staff when MSUD formula was brought to his bedside for him at the psychiatric holding unit. His examination was notable for ataxia and waxing and waning attention. When asked about his formula, he was at his appropriate cognition to confirm the reason he needs it. He endorsed seeing “mean” and “scary” things. He was given MSUD formula in the psychiatric holding unit without complications and the community hospital arranged for plasma amino acids to be performed by the tertiary facility immediately. Plasma amino acid resulted on the subsequent day with isoleucine 357 μmoles/L (normal: 30–108), leucine 1843 μmoles/L (normal 66–170), and valine 979 μmoles/L (normal: 119–336). In addition, the alanine, alanine/lysine ratio, and glutamine levels were normal; thus, suggesting that ammonia and lactate elevations were not the cause of his hallucinations.

Arrangement was made for the patient to be transferred to his metabolic center once a safe transfer could be facilitated. He was started on dextrose containing IV fluid immediately prior to transfer to our facility by the transport team. Given his elevation of leucine to approximately ten times the upper limit of normal, we considered initiation of renal replacement therapy which has been shown to be effective in treating acutely decompensated MSUD [[13], [14], [15], [16]]. While the hemodialysis is effective, it is not a benign procedure with potential adverse outcomes [17]. Given that our patient was protecting his airway and continued to have ability to interact with the care team, the decision was made to initiate combination of enteral and parenteral nutrition which has also been shown to be effective in reduction of plasma leucine levels [5,18].

**Fig. 1:**
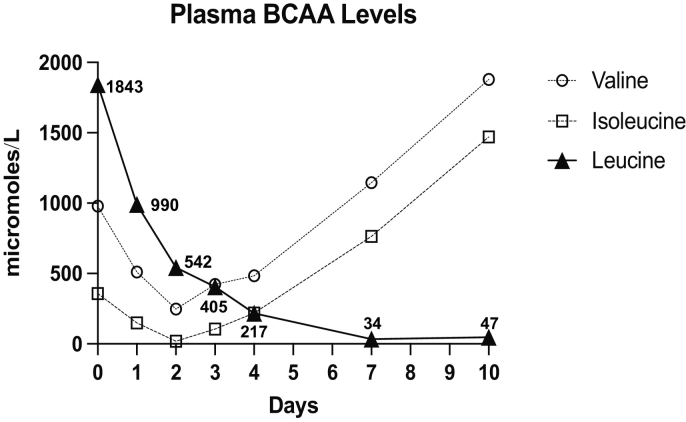
Plasma amino acid analysis showing branched chain amino acids from the patient collected during his hospitalization at our facility. Numbers next to the filled triangle notes concentration of plasma leucine in micromoles/L.

**Fig. 2:**
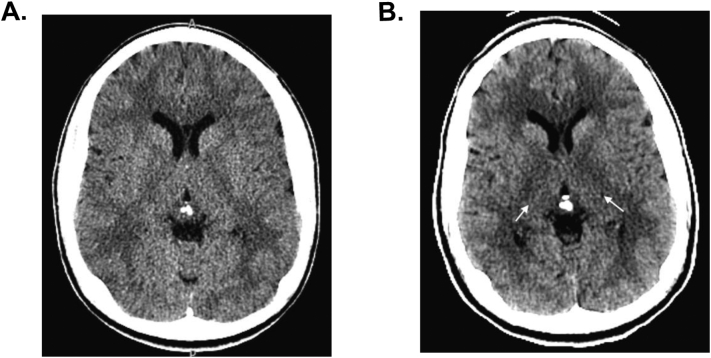
**CT scan of the patient's head.** A) Baseline axial CT image through the cerebral deep gray nuclei shows symmetric, normal gray-white matter distinction without focal cerebral lesions. B) Axial CT image obtained during the recent hospitalization. Image through cerebral deep gray nuclei shows subtle hypodensity infiltrating the thalami, internal capsules, and adjacent globi pallidi (arrows) consistent with edema in the setting of metabolic decompensation.

Upon arrival, he was continued on dextrose containing IV fluid (D10 normal saline at twice maintenance) and started on intralipids (2 g/kg). Simultaneously, he was started on branched-chain amino acid-free formula Ketonex-2 (Abbott) (providing 1.6 g/kg protein) with supplementation of L-isoleucine (20 mg/kg) and L-valine (20 mg/kg). Serial amino acid levels were obtained to trend his leucine levels (Fig. 1). In addition, his urine ketone levels were monitored until ketones cleared, which occurred within 36 h of beginning IV fluids. His CT scan was reviewed by a pediatric neuroradiologist who interpreted the images as mild edema in the globi pallidi and thalami (Fig. 2A and B). More widespread mild hypoattenuation in the cerebral, cerebellar, and brainstem white matter may indicate additional regions of recurrent white matter edema. Patient remained hospitalized until his leucine levels were reduced to the normal ranges, which required approximately five days (Fig. 1) (normal: 66 to 170 μmol/L), and his cognition and behavior returned to baseline. He was dischared with a restroation of his baseline diet (1.6 g/kg medical protein; 0.3 g/kg natural protein; 29 mg/kg leucine) and freqent outpaitent follow up. Outpatient follow up utilized telemedicine to provide at home education on food choices and reeducation of formula mixing.

## 3 Discussion

We report a known patient with MSUD who presented to the hospital due to acute hallucinations and ataxia secondary to metabolic decompensation of MSUD who had delayed access to care due to a disjointed health care system. Despite the possibility of acute MSUD decompensation mentioned in early documentation by an adult physician, patient was admitted for psychiatric evaluation and received multiple rounds of medications for his agitation which were only partially effective. While the care was delayed, the patient was able to receive appropriate attention once the astute community hospital nurse reached out to the Children's National Hospital transport team to determine who might be able to help them with questions about metabolic formula. From there, the transport team paged the on-call genetics team who immediately recognized the patient's name and urgently delivered appropriate metabolic formula to the community hospital.

Upon further review, it was found that the patient had dental procedures the week prior without notifyign the metabolic center and had been transported to a hospital where he had not been previously evaluated. This was due to a COVID-19 pandemic diversion at the two closer hospitals, which had electronic medical alerts to flag for his metabolic disorder. In addition, it was found that a caring relative had brought his metabolic formula for him to the hospital, but it had not been used since there were not “directions on the can”.

MSUD is one of more than 1400 rare inborn errors of metabolism (IEMs) caused by inherited defects in biochemical pathways [19]. While the individual reported incidence of IEMs ranges from 1 in 10,000 to 1 in 1 million, collectively the incidence can be as high as 1 in 800 to 1 in 2500 [19]. Advances in diagnosis and treatment have improved the prognosis and survival of patients with IEMs and more than 90% will survive beyond the age of 20 years [[19], [20], [21]]. Thus, this case highlights the importance of expanding training in rare metabolic disorders to adult care providers. In addition, this case also highlights the important secondary development of neurocognitive and psychiatric manifestations of MSUD due to prolonged exposure to leucine [9,11,12]. As MSUD patients are prone to development of neurocognitive and psychiatric morbidities, it is important to establish early evaluation and therapeutic plan with psychiatrists in the community to optimize their access to mental health care and quality of life.

While improved prognosis and survival have improved quality of life for patients and their families, increased survival has created new challenges such as appropriate transition of care, as demonstrated in this case. Appropriate transition of care requires adolescent IEM patients shifting care from metabolic pediatricians to metabolic physicians specialized in treating adults [19,22]. Furthermore, development and coordination of a multidisciplinary team for each individual IEM becomes critical to ensure that patients with IEMs achieve good quality of life as adults [19,23]. While the implementation of a multidisciplinary team for IEM is extremely important, patients and families must also become empowered to take an active role in managing their condition (Table 1) [19,23].

**Table 1:** List of potential interventions to be implemented in patients with IEMs transitioning care.

	Interventions	Strategies	Goals
Patient	Disease specific education/skills training	• One to one teaching • Printed material • Adolescent friendly website • Group sessions	• Improve understanding • Improve self-management skill • Increase autonomy • Identify high risk situations (i.e. dental procedures)
	Disease identifier	• Wallet cards • Medical alert card • Medical bracelet	• Improve disease awareness • Early initiation of treatments during illness • Improve early contact with medical team
Medical Team	Transition coordinator	• Point of contact for patients • Create point of contacts for extended family members	• Improve continuity of care • Provide transition planning • Prepare patients • Increasing availability of family members for communication support
	Transitional clinic	• Clinic attendance by pediatric and adult physicians • Expand visits to extended family members	• Introduction to transition • Improve continuity of care • Improve information exchange/sharing • Increasing availability of family members for support
	Telemedicine	• Telephone visits • Video visits (Zoom, Facetime, WebEx, Microsoft Teams)	• Improve clinic attendance/follow up through utilization of telemedicine • Improve follow up in patients with limited transportation • Improve follow up for patients that live far from the clinic
Health System	Dedicated emergency treatment center	• Stable hospital for admission • Notify EMS about the special patient	• Consistent, acute treatments • Appropriate transport of patients to selected facilities
	Notify the treatment team for admission	• Insurance company to notify the outpatient team	• Initiate appropriate care and/or transfer if needed
	Enhanced follow ups	• Automatic telephone reminders and/or calls if the appointment is missed	• Improve the rates of follow up and encourage their follow up

Transition of care poses significant challenges for our patients and the clinical team, and the need for improved transition will likely continue to increase with improved diagnostic and treatment methods. It is not only unique to the IEM patient population, but it is applicable to all conditions that require chronic lifelong care [[22], [23], [24], [25], [26]]. There are a number of potential interventions and systems that have been proposed including but not limited to creation of continuing medical education (CME) programs, education and training healthcare professionals from all disciplines and non-healthcare professionals such as teachers and caregivers, use of smart cards to provide immediate access to a patient's medical history and information for emergency use, and/or mobile apps [[22], [23], [24], [25], [26]].

Given that acute decompensations of IEMs are a medical emergency, and the patients require immediate treatment, we propose interventions that are readily available such as use of medical alert bracelets, creating a calling plan with extended family, and/or identifying local hospitals that can be requested in emergency situations.

## Declaration of Competing Interest

The authors report no subject specific conflicts of interest.

## Data Availability

No data was used for the research described in the article.

## References

[1] Ogier de Baulny H., Saudubray J.M. (2002). Branched-chain organic acidurias. Semin. Neonatol..

[2] Strauss K.A., Puffenberger E.G., Carson V.J., Adam M.P., Ardinger H.H., Pagon R.A., Wallace S.E., LJH Bean, Gripp K.W., Mirzaa G.M., Amemiya A. (1993). GeneReviews((R)).

[3] de Lonlay P., Posset R., Mutze U., Mention K., Lamireau D., Schiff M., Servais A., Arnoux J.B., Brassier A., Dao M. (2021). Real-world management of maple syrup urine disease (MSUD) metabolic decompensations with branched chain amino acid-free formulas in France and Germany: a retrospective observational study. JIMD Rep..

[4] Servais A., Arnoux J.B., Lamy C., Hummel A., Vittoz N., Katerinis I., Bazzaoui V., Dubois S., Broissand C., Husson M.C. (2013). Treatment of acute decompensation of maple syrup urine disease in adult patients with a new parenteral amino-acid mixture. J. Inherit. Metab. Dis..

[5] Morton D.H., Strauss K.A., Robinson D.L., Puffenberger E.G., Kelley R.I. (2002). Diagnosis and treatment of maple syrup disease: a study of 36 patients. Pediatrics.

[6] Frazier D.M., Allgeier C., Homer C., Marriage B.J., Ogata B., Rohr F., Splett P.L., Stembridge A., Singh R.H. (2014). Nutrition management guideline for maple syrup urine disease: an evidence- and consensus-based approach. Mol. Genet. Metab..

[7] Blackburn P.R., Gass J.M., Vairo F.P.E., Farnham K.M., Atwal H.K., Macklin S., Klee E.W., Atwal P.S. (2017). Maple syrup urine disease: mechanisms and management. Appl. Clin. Genet..

[8] Mutze U., Garbade S.F., Gramer G., Lindner M., Freisinger P., Grunert S.C., Hennermann J., Ensenauer R., Thimm E., Zirnbauer J. (2020). Long-term outcomes of individuals with metabolic diseases identified through newborn screening. Pediatrics.

[9] Strauss K.A., Carson V.J., Soltys K., Young M.E., Bowser L.E., Puffenberger E.G., Brigatti K.W., Williams K.B., Robinson D.L., Hendrickson C. (2020). Branched-chain alpha-ketoacid dehydrogenase deficiency (maple syrup urine disease): treatment, biomarkers, and outcomes. Mol. Genet. Metab..

[10] Carecchio M., Schneider S.A., Chan H., Lachmann R., Lee P.J., Murphy E., Bhatia K.P. (2011). Movement disorders in adult surviving patients with maple syrup urine disease. Mov. Disord..

[11] Muelly E.R., Moore G.J., Bunce S.C., Mack J., Bigler D.C., Morton D.H., Strauss K.A. (2013). Biochemical correlates of neuropsychiatric illness in maple syrup urine disease. J. Clin. Invest..

[12] Shellmer D.A., DeVito Dabbs A., Dew M.A., Noll R.B., Feldman H., Strauss K.A., Morton D.H., Vockley J., Mazariegos G.V. (2011). Cognitive and adaptive functioning after liver transplantation for maple syrup urine disease: a case series. Pediatr. Transplant..

[13] Atwal P.S., Macmurdo C., Grimm P.C. (2015). Haemodialysis is an effective treatment in acute metabolic decompensation of maple syrup urine disease. Mol. Genet. Metab. Rep..

[14] Aygun F., Aygun D., Erbek Alp F., Zubarioglu T., Zeybek C., Cam H. (2018). The impact of continuous renal replacement therapy for metabolic disorders in infants. Pediatr. Neonatol..

[15] Porta F., Peruzzi L., Bonaudo R., Pieretti S., Busso M., Cocchi E., Conio A., Pagliardini V., Spada M. (2018). Differential response to renal replacement therapy in neonatal-onset inborn errors of metabolism. Nephrology (Carlton).

[16] Puliyanda D.P., Harmon W.E., Peterschmitt M.J., Irons M., Somers M.J. (2002). Utility of hemodialysis in maple syrup urine disease. Pediatr. Nephrol..

[17] Kumar A., Cage A., Dhar R. (2015). Dialysis-induced worsening of cerebral edema in intracranial hemorrhage: a case series and clinical perspective. Neurocrit. Care..

[18] Scott A.I., Cusmano-Ozog K., Enns G.M., Cowan T.M. (2017). Correction of hyperleucinemia in MSUD patients on leucine-free dietary therapy. Mol. Genet. Metab..

[19] Stepien K.M., Kiec-Wilk B., Lampe C., Tangeraas T., Cefalo G., Belmatoug N., Francisco R., Del Toro M., Wagner L., Lauridsen A.G. (2021). Challenges in transition from childhood to adulthood care in rare metabolic diseases: results from the first multi-center European survey. Front. Med. (Lausanne).

[20] Blum R.W. (1995). Transition to adult health care: setting the stage. J. Adolesc. Health.

[21] Schwarz M., Wendel U. (2005). Inborn errors of metabolism (IEM) in adults. A new challenge to internal medicine (part 2). Med. Klin. (Munich).

[22] Lampe C., McNelly B., Gevorkian A.K., Hendriksz C.J., Lobzhanidze T.V., Perez-Lopez J., Stepien K.M., Vashakmadze N.D., Del Toro M. (2019). Transition of patients with mucopolysaccharidosis from paediatric to adult care. Mol. Genet. Metab. Rep..

[23] Crowley R., Wolfe I., Lock K., McKee M. (2011). Improving the transition between paediatric and adult healthcare: a systematic review. Arch. Dis. Child..

[24] Lopez K.N., O’Connor M., King J., Alexander J., Challman M., Lovick D.K., Goodly N., Smith A., Fawcett E., Mulligan C. (2018). Improving transitions of care for young adults with congenital heart disease: mobile app development using formative research. JMIR Form. Res..

[25] Pedersen M., Hoybye C. (2021). An adapted model for transition to adult care in young adults with prader-willi syndrome. J. Clin. Med..

[26] Van Lierde A., Menni F., Bedeschi M.F., Natacci F., Guez S., Vizziello P., Costantino M.A., Lalatta F., Esposito S. (2013). Healthcare transition in patients with rare genetic disorders with and without developmental disability: neurofibromatosis 1 and Williams-Beuren syndrome. Am. J. Med. Genet. A.

